# Development of running is not related to time since onset of independent walking, a longitudinal case study

**DOI:** 10.3389/fnhum.2023.1101432

**Published:** 2023-02-16

**Authors:** Margit M. Bach, Coen S. Zandvoort, Germana Cappellini, Yury Ivanenko, Francesco Lacquaniti, Andreas Daffertshofer, Nadia Dominici

**Affiliations:** ^1^Department of Human Movement Sciences, Faculty of Behavioural and Movement Sciences, Amsterdam Movement Sciences & Institute of Brain and Behavior Amsterdam, Vrije Universiteit Amsterdam, Amsterdam, Netherlands; ^2^Laboratory of Neuromotor Physiology, Istituto di Ricovero e Cura a Carattere Scientifico Fondazione Santa Lucia, Rome, Italy; ^3^Department of Systems Medicine, Center of Space Biomedicine, University of Rome Tor Vergata, Rome, Italy

**Keywords:** children, development, running, clustering, muscle synergies, neuromuscular control, kinematics

## Abstract

**Introduction:**

Children start to run after they master walking. How running develops, however, is largely unknown.

**Methods:**

We assessed the maturity of running pattern in two very young, typically developing children in a longitudinal design spanning about three years. Leg and trunk 3D kinematics and electromyography collected in six recording sessions, with more than a hundred strides each, entered our analysis. We recorded walking during the first session (the session of the first independent steps of the two toddlers at the age of 11.9 and 10.6 months) and fast walking or running for the subsequent sessions. More than 100 kinematic and neuromuscular parameters were determined for each session and stride. The equivalent data of five young adults served to define mature running. After dimensionality reduction using principal component analysis, hierarchical cluster analysis based on the average pairwise correlation distance to the adult running cluster served as a measure for maturity of the running pattern.

**Results:**

Both children developed running. Yet, in one of them the running pattern did not reach maturity whereas in the other it did. As expected, mature running appeared in later sessions (>13 months after the onset of independent walking). Interestingly, mature running alternated with episodes of immature running within sessions. Our clustering approach separated them.

**Discussion:**

An additional analysis of the accompanying muscle synergies revealed that the participant who did not reach mature running had more differences in muscle contraction when compared to adults than the other. One may speculate that this difference in muscle activity may have caused the difference in running pattern.

## 1. Introduction

Independent walking is a major developmental milestone for children. In typically developing children it commonly occurs between 9 and 15 months of age (Piper and Darrah, 1994; Storvold et al., 2013). While most parents can recall at what age their children started walking independently, almost none of them can put a finger on when the children started running. One reason for this might be the difficulty to tell walking and running apart. This is not so in adults’ locomotion, where even a still picture may serve to distinguish walking from running. Obviously, the presence of a phase of flight can do the job, i.e., when no leg touches the ground. In very young children, discriminating walking from running is often not that straight-forward. Early running may appear as fast walking which raises the question on which parameters these two locomotory states really differ.

In a previous work we studied 5-9-year-old children (Bach et al., 2021a). There we realized that classical measures for classifying walking and running mostly fail. Neither the presence of a flight phase nor the phase relation of energetics was sufficient to distinguish mature from immature running patterns in these children. We suggested using a ‘shotgun’ approach involving a large set of kinetic and kinematic parameters with subsequent principal component analysis (PCA) and hierarchical clustering that allowed for separating the degree of maturity of walking and running with great success.

As it turns out, there is no immediate agreement between the chronological age and the maturity of treadmill walking and running patterns (Bach et al., 2021a). Yet, the amount of walking experience clearly influences the walking pattern and that improves with practice (Sutherland et al., 1980; Forssberg, 1985; Cheron et al., 2001; Ivanenko et al., 2005). Be it the recovery of mechanical energy, the external work, or the inter-segmental kinematic coordination, all these features gradually evolve toward those of adults when walking experience increases (Ivanenko et al., 2004; Ivanenko et al., 2007a). Not only do joint kinematics and kinetics improve progressively (Hallemans et al., 2006), but also the duration of electromyographic activity of the gastrocnemius medialis muscle is reduced (Cappellini et al., 2016). We believe that the (gain of) walking experience also influences the development of running which ultimately tends toward the mature pattern observed in adults. Very recently, it has been shown that the motor control of running is influenced by motor exploration and learning (Bach et al., 2021b). As such, it seems quite likely that developmental characteristics of walking are also mirrored in the development of running.

Tackling such commonalities is a challenge, which may fail when following more traditional routes in studying locomotion, namely from either a sole neuromuscular (e.g., Ivanenko et al., 2006) or a sole biomechanics perspective (e.g., Rozumalski et al., 2015; Liu et al., 2022). We advocate combining both perspectives as several recent studies suggest their interdependence during infancy (Forssberg, 1985; Dominici et al., 2011; Cappellini et al., 2016; Dewolf et al., 2020; Bekius et al., 2021). At the onset of independent locomotion, walking and running may overlap so strongly for their neural and biomechanical control that some consider walking and running in infants not as distinct modes of locomotion as they are in adults (Vasudevan et al., 2016; Dewolf et al., 2020). If walking and running are intertwined when infants learn to walk, then at which moment will they “separate” as much as in adults?

Answering the relation between the onset of independent walking and the development of running requires longitudinal assessments spanning several years. To quantify the influence of time since onset of independent walking, one must assess participants at the very onset of independent walking (in fact assesement have to start even before that). And recordings must be frequent enough to properly sample the development of running. We monitored two typically developing children for about three years after their first independent walking steps. We conducted seven experimental sessions during each of which we guaranteed more than 100 running strides when recording leg and trunk 3D kinematics and electromyography (EMG). Using the aforementioned shotgun method that encompasses kinematics and neuromuscular data, we investigated the earliest development of running. Possible mechanisms underlying the coordinated locomotion were explored through muscle synergy analysis and by integrating some of the corresponding outcome parameters in the shotgun approach. We expected this approach to allow for determining the degree of maturity also in very young children who just learned / are learning to run. We expected the development of running maturity to be similar, if not identical, to the onset of independent walking when stratifying its time course.

## 2. Materials and methods

### 2.1. Participants

We recruited two children and five adults. The two children (1 male/1 female) were recruited before taking their first independent steps as part of a larger study (Zandvoort et al., 2022). The adult participants (4 male/1 female, 30-45 years old) were recruited by word-of-mouth as part of a previous study (Cappellini et al., 2006). Both the adults and the legal guardians of both children gave written informed consent in compliance with the Declaration of Helsinki. The inclusion of the children was approved by The Scientific and Ethical Review Board of the Faculty of Behavioural & Movement Sciences, Vrije Universiteit Amsterdam, Netherlands (File number: VCWE-2016-082). The inclusion of adults was in accordance with the procedures of the Ethics Committee of the Santa Lucia Institute, Rome, Italy (Prot. CE-AG4-PROG.99-155).

The first recording session of each child participant took place within 9 days of taking at least four consecutive steps without support. The time of first indpendent steps were relayed by the parents to the researchers.

### 2.2. Setup

**FIGURE 1:**
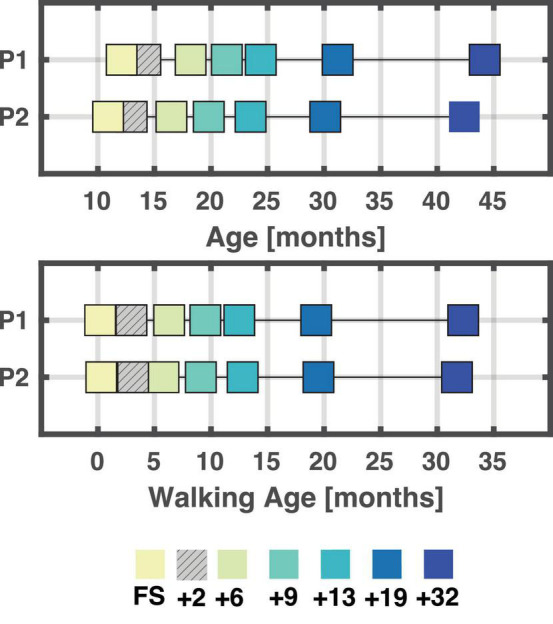
Overview of age and walking age (time since onset of walking) for each session/child. A total of seven sessions were recorded for each child. At the end, six sessions were analyzed for each child, and these were matched based on walking age (bottom plot) in months. A few weeks separate the different sessions between P1 and P2. +2 is hatched as not analysed due to poor data quality. FS: First steps, +2, +6, +9, +13, +19, +32 refers to the number of months since first steps, i.e., time since onset of independent walking.

Seven sessions were recorded from first steps (FS) to ∼ 32 months after onset of independent walking for each of the two children (P1 and P2). The initial plan was to record each child every three months from their first independent steps until one year after onset of independent walking with a follow-up every six months from that timepoint. As this was not achieved with the first child, we matched the second child to the spacing of the recordings of the first child. As sketched in Figure 1, the following sessions were recorded: first steps session (FS), 2 months after the FS (denoted +2), as well as 6 months (+6), 9 months (+9), 13 months (+13), 19 months (+19), and finally 32 months after FS (+32).

The experiments consisted of locomoting overground and on a pediatric treadmill (N-Mill 60 × 150 cm, Motek Medical BV, Amsterdam, the Netherlands) with either no support or trunk/hand support. During the first session (FS), only walking was recorded. During the subsequent sessions, we recorded both walking and fast walking and/or running. Children were tasked to move from one end of the lab to the other end. They were instructed to either walk or run, sometimes enticed with toys or food. When the child was instructed to run, but the speed was between their normal running and walking speed, then the trial was noted as “fast walking”. For sessions +6, +9, +13, +19, and +32, only the trials labelled as fast walking or running were retained for further analysis.

All children sessions, except one, were recorded in the BabyGaitLab of the Department of Human Movement Sciences at the Vrije Universiteit Amsterdam, The Netherlands; the remaining session was recorded at the clinical gait laboratory of the Department of Rehabilitation Medicine at the Amsterdam UMC (location VUmc). The adults were recorded in the laboratory of the Santa Lucia Foundation, Rome, and included in a previous publication (Cappellini et al., 2006).

The children were barefoot during all recordings and wore only diapers or underpants. When locomoting overground, they were encouraged by researchers/parents to walk/run in a straight line. Sometimes they were supported by handhold. When on the treadmill, the speed was adjusted to a comfortable speed and type of locomotion (walk or run). On the treadmill, the children were supported on trunk or by handhold by the researcher/parent. Adults were running at 7 and 9 km/h on a treadmill (EN-MILL, 3446.527, Bonte Zwolle BV, Netherlands) wearing shoes.

### 2.3. Data acquisition

#### 2.3.1. Toddlers

During each session for P1 and P2, bilateral 3D kinematics was recorded using reflective markers and Vicon motion capture system (Oxford, UK) with 10 (12 for the session recorded at VUmc) infrared cameras affixed to the ceiling, sampled at 100 Hz and one (four for the session recorded at VUmc) video camera (Vicon camera Oxford, UK) sampled at 100 Hz (50 Hz for the session recorded at VUmc). Reflective markers (14 mm) were placed bilaterally on the acromion (SHO), iliac crest (IL), greater trochanter (GT), lateral femur epicondyle (LE), lateral malleolus (LM) and fifth metatarsal (VM). For each session, a static trial was recorded where the participant was standing still to be used for correction of the joint angles. We recorded electromyography (EMG) bilaterally from the following 16 muscles: tibialis anterior (TA), medial gastrocnemius (MG), lateral gastrocnemius (LG), soleus (SOL), rectus femoris (RF), vastus medialis oblique (VMO), vastus lateralis oblique (VLO), semitendinosus, biceps femoris (BF), tensor fascia latae (TFL), gluteus maximus (GLM), erector spinae level L2 (ES), latissimus dorsi, trapezius, deltoid, and biceps brachii. EMG was recorded using mini-golden reusable surface EMG disc-electrode pairs (15-mm-diameter electrodes, acquisition area of 4 mm^2^), placed at the approximate location of the muscle belly on the cleaned skin, with interelectrode spacing of ∼1.5 cm. The placement followed the SENIAM recommendations (Hermens et al., 1999), and were sampled at 2 kHz. Movement artifacts were minimized by fixating the electrodes and wireless EMG sensors to the leg using elastic gauzes. EMG was recorded with a wireless system (Mini wave plus, Zerowire; Cometa, Bareggio, Italy) and saved in Nexus software as backup. EMG recordings included an online bandpass filter 10 Hz-1 kHz. For each session, we also recorded electroencephalogram (EEG) using pre-gelled caps (ANT neuro, Hengelo, The Netherlands) which could not be included in the analysis due to too many artefacts.

The pediatric treadmill recorded vertical ground reaction forces with a sampling frequency of 1 kHz. For each session, the anthropometrics of the child was measured and recorded, such as the total length, the weight measured by weighing scales *m*, the body weight measured by treadmill *bw*_treadmill_, and the length and circumference of the main body segments (Schneider and Zernicke, 1992). The segment lengths estimated from the static trials were used to determine leg length.

When running on the treadmill, children were supported on the trunk or by handhold by the researcher/parent. The amount of body weight support (BWS) provided to the children during treadmill trials were estimated as the percentage reduction of the mean vertical forces compared to *bw*_treadmill_. More than 30% of BWS may result in altered foot trajectories and temporal patterns of the muscle synergies in toddlers walking (Dominici et al., 2007; Kerkman et al., 2022). Thus, only strides with less than 30% BWS were retained for further analysis (∼22 and ∼28% of strides were removed for P1 and P2, respectively).

#### 2.3.2. Adults

Data acquisition has been described previously in Cappellini et al. (2006). In brief, we used reflective markers (14 mm) and Vicon motion capture system (Vicon camera Oxford, UK, sampling at 100 Hz) with 9 infrared cameras spaced around the treadmill to record bilateral 3D kinematic. Reflective markers were placed bilaterally on SHO, IL, GT, LE, LM, heel, and VM; these are the same anatomical locations as used for P1 and P2 (except for the heel marker). The following 32 muscles were recorded unilaterally: TA, flexor digitorum brevis, LG, MG, SOL, peroneus longus, VLO, VMO, RF, sartorius, BF, semitendinosus, adductor longus, TFL, GLM, gluteus medius, external oblique, internal oblique, latissimus dorsi, iliopsoas, rectus abdominis, erector spinae recorded at T1, T9, and L2 (ES), biceps brachii, triceps brachii, deltoideus (anterior and posterior portions), trapezius (inferior and superior portions), sternocleidomastoid, and splenius using Delsys electrodes (model DE2.1, Delsys, Boston, MA). The signals were amplified, filtered (20-450 Hz), and sampled at 1 kHz. Height and weight were recorded for all participants. Leg lengths were not recorded but could be inferred from the 3D kinematics.

### 2.4. Data analysis

#### 2.4.1. Kinematics and gait parameters

Foot contact and foot-off were manually determined for both sides by visual inspection using digitial video recordings and the foot marker trajectories from the Nexus software (Vicon, Oxford, UK) in the children. For the adults, foot contact was defined as the local minima of the heel marker and foot off as the lift-off of the VM marker by 2 cm from the minimum detected at stance (Cappellini et al., 2006). Strides with jumping, dragging etc. were excluded from further analysis as was gait initiation and termination. Flight phases and double support phases were determined based on right and left foot contact and foot off. The Froude number (Fr) is a dimension-less parameter suitable for the comparison of locomotion in subjects of different size (Alexander and Jayes, 1983). The Froude number was computed for all gait cycles based on the mean velocity of the horizontal IL marker (*v*), leg length (*l*), and the gravitational constant (*g*) using: *Fr* = *v*^2^/(*g* ⋅ *l*). Kinematic parameters were calculated based on the 3D kinematics of the lower legs and trunk. The body was modeled as an interconnected chain of rigid segments: SHO-IL for the trunk, IL-GT for the pelvis, GT-LE for the thigh, LE-LM for the shank, and LM-VM for the foot. The main limb axis was defined as the virtual line connecting GT and VM. Joint and elevation angles were generated accordingly. A total of 101 parameters were estimated for each gait cycle using a custom-written algorithm (Dominici et al., 2012; Bach et al., 2021a) to provide a comprehensive quantification of locomotor patterns. They can be functionally split into themes such as temporal features, limb endpoint trajectory, stability, joint and segment angles, joint and segment angular velocities, intra- and interlimb coordination, intersegmental coordination, and pendulum mechanism. Parameters that were directly influenced by body size were normalized to leg length. For a detailed list we refer to Supplementary material 1. All parameters were visually inspected for outliers due to experimental errors (e.g., partially missing markers) and, if the errors were present, the stride was removed from further analysis (∼4 and ∼8% of total recorded strides for P1 and P2, respectively).

#### 2.4.2. Electromyography and muscle synergies

Of the recorded muscles, the following 11 (bilateral for the children, unilateral for the adults) muscles TA, MG, LG, SOL, RF, VMO, VLO, BF, TFL, GLM, and ES were retained for further analysis. EMG data were visually inspected, and artifacts were removed using a custom-written burst-detection algorithm (Bach et al., 2021b; Zandvoort et al., 2022). After high-pass (2nd-order bidirectional Butterworth filter at 20 Hz; De Luca et al., 2010; Willigenburg et al., 2012; Bach et al., 2021b) and notch filtering (2^nd^-order bi-directional Butterworth around k⋅50 Hz, k = 1,…,10, with half-bandwidth of 0.5 Hz), the EMG data were rectified using the modulus of the analytic signal and finally low-pass filtered (bi-directional 2^nd^-order Butterworth filter at 10 Hz) to obtain the corresponding EMG envelopes (Dominici et al., 2011; Oliveira et al., 2016; Bekius et al., 2021). These envelopes were time-normalized to 200 samples per gait cycle computed relative to the ipsilateral foot contact.

Before applying the muscle synergy analysis, the amplitude of the EMG activity was normalized to the mean activity for each muscle, interpolated in case of missing values (for a maximum of 50% of the stride) within one stride, and finally concatenated for each session in a [#strides × #samples] × #muscles matrix ([n × 200] × 11). Post-hoc analysis of the interpolation revealed that the mean interpolation was 4.5% for P1 (range: 0.5%-19.5%) and 4.6% for P2 (range: 0.5%-34%) in approximately 22% and 22.6% of the total number of strides, respectively. The gaps were not necessarily consecutive. No interpolation was done on the adult data. Muscle synergies were calculated using weighted non-negative matrix factorization (WNMF) algorithm (with a maximum of 2.10^6^ iterations, and a completion threshold of 10^–6^) to account for missing strides (Li and Ngom, 2013; Goudriaan et al., 2022). There were missing data in few strides where no EMG was recorded for one or two muscles (Li and Ngom, 2013; Shuman et al., 2019; Goudriaan et al., 2022). WNMF decomposes the original EMG matrix into a small set of temporal activation patterns (*C*) and weighting coefficients (*W*):


$$E\,M\,G=\sum_{i=1}^{N}C_{i}\cdot W_{i}+\epsilon ,N\leq \#\,\mathrm{muscles}$$

With ϵ denoting the residual error. To assess the quality of the reconstruction, the reconstruction accuracy (Zandvoort et al., 2019; Kerkman et al., 2020; Bach et al., 2021b; Kerkman et al., 2022) was determined using the Frobenius norm of the residuals


$$R\,A=1-\frac{{||E\,M\,G-\left(W\cdot C\right)||}_{F}}{{||E\,M\,G||}_{F}}$$

We determined the number of synergies for further analysis via the “best linear fit” proposed by Cheung et al. (2005). For this, one computes the mean squared error for each linear fit of the reconstruction quality for first 1-10 synergies, then 2-10 until calculated for 9-10 synergies. When the mean squared error drops below 10^–4^ the reconstruction quality is said to plateau defining the number of synergies to retain. To align the number of synergies across sessions, the best linear fit method was applied to each session of the children and the adults, respectively, and the median number of synergies across these 13 sessions that fulfilled this criterion was chosen, thus avoiding a bias towards the mean in the case of outliers.

The output of the WNMF is not ranked and as such post-hoc sorting has to be applied to compare synergies across sessions. To do so, the weighting coefficients were grouped using hierarchical clustering during which we ensured that the maximum number of clusters corresponded to the maximum number of synergies (i.e., a maximum of three clusters were allowed with a three-synergy solution). For the synergy analysis, the grand average of all strides for each synergy was determined per session.

To quantify differences in the duration of the temporal activation patterns of the muscle synergies, we estimated the full width at half-maximum (FWHM) per activation pattern and stride. Here we first subtracted the minima of the activity patterns – in the case of several peaks, the FWHM was calculated for the main peak, i.e., the peak with the highest amplitude and in case of boundary peaks, an assumption was made that the shape of the peak was symmetric (Cappellini et al., 2006). Timing differences were determined via the center-of-activity (CoA) per activation pattern and stride (Yakovenko et al., 2002; Labini et al., 2011; Sylos-Labini et al., 2014). The CoA is particularly useful when multiple peaks are present or when low activity does not allow for identifying a single peak. CoA also makes the comparison across sessions feasible. Here, we defined it as


$$C\,o\,A={\text{tan}}^{-1}\,\left[\frac{\sum_{t=1}^{200}\left(\cos\theta_{t}\cdot E\,M\,G_{t}\right)}{\sum_{t=1}^{200}\left(\sin\theta_{t}\cdot E\,M\,G_{t}\right)}\right]$$

where, θ denotes an angle that varies between 0-360° corresponding to 0-100% of the gait cycle (*t* = 200 samples). The FWHM and the CoA for the extracted synergies were retained and added to the list of gait parameters for further analysis to have a spatial as well as temporal measure for the activation patterns of the muscle synergies (Supplementary material 1).

#### 2.4.3. PCA and clustering

We sought to quantify how running develops over time from the first independent steps. Strides from trials in which the children were instructed to run (that were labelled either fast walking or running) were included. We chose for this ‘blind’ approach as our previous research revealed that in very young children the presence of a flight phase is not a solid indicator for the presence of running (Bach et al., 2021a). We employed principal component analysis (PCA) in combination with clustering of several parameters extracted from the kinematics and muscle synergies for all strides (Bach et al., 2021a). PCA served to reduce covariation between parameters and clustering to find unbiased classification.

For every participant, parameters were combined in a [(number of sessions × number of strides) × number of parameters] matrix [1730 × 107] and z-scored prior to PCA, see Supplementary material 1 for a full overview of the parameters. The z-scoring was applied to ensure that all parameters could potentially contribute to the same degree in the PCA (if the variance differs between parameters it may cause a bias in the PCA-ranking). We selected the three leading principal components (PCs) and included them in the clustering, as this turned out sufficient for our classification purposes (Courtine et al., 2009; Dominici et al., 2012; Friedli et al., 2015; Phinyomark et al., 2015; Bach et al., 2021a). The degree to which the different parameters influence the first three PCs can be given by their $l\,o\,a\,d\,i\,n\,g\,s=\upsilon \cdot \sqrt{\lambda }$, where υ denotes the eigenvector of a PC and λ its eigenvalue. We considered a parameter as a strong contributor if the corresponding loadings exceeded the 95% confidence interval $C\,I_{95}=1.96/\sqrt{n}$ where *n* = 107 parameters.

Finally, we applied hierarchical clustering with correlation distance (Bach et al., 2021a). We first built a dendrogram (Milligan, 1980; Xu and Wunsch, 2005; Murtagh and Contreras, 2011) using average links (unweighted pair group method with arithmetic mean). The cophenetic correlation coefficient was determined (CCC; Sokal and Rohlf, 1962) to establish the degree of fit of the clustering technique. The Calinzki-Harabasz stopping rule (Milligan and Cooper, 1985) and visual inspection were utilized in unison to determine the optimal number of clusters, with the inspection focusing on categorization of first steps walking and running and the classification of mature and immature running. We distinguished mature from immature locomotion by computing the average pairwise correlation distance from every stride belonging to a distinct cluster to the adults running. Put differently, the average pairwise correlation distance served as a measure for gait maturity with the adult gait pattern as reference.

### 2.5. Statistics

Means and standard deviations are provided unless otherwise specified. To investigate whether the dimensionless speed Fr and the FWHM of the muscle activation patterns were different between sessions for each participant and comparable to the adults, we used a non-parametric test, the Kruskal-Wallis test, as the data were not normally distributed, confirmed using a Kolmogorov-Smirnov goodness-of-fit hypothesis test. If a statistically significant effect was found, a Bonferroni correction was applied to account for multiple comparisons. With 7 mixed within-between participants (6 sessions for the toddlers and 1 session for the adults), the used significance threshold was α=0.05/7=0.007.

## 3. Results

Our child participants were comparable in terms of age as well as time in months since first independent steps. P2 started walking at 10.6 months whereas P1 started walking at 11.9 months. Both children were relatively early walkers. The median age of independent walking lies between 11.4 months (Piper and Darrah, 1994) and 13.0 months (Storvold et al., 2013). The different sessions were comparable and within a few weeks of each other in terms of walking age. All results were ordered based on the walking age to investigate the influence of walking age.

The first sessions, the FS sessions, were recorded within 9 days of when the children performed at least four independent strides, and for these sessions only walking was recorded. In the subsequent sessions, the children were instructed to either walk or run, but only strides from trials when instructed to run (that were labelled as fast walking or running during the experiment, see *2.2 Setup* for further details) were analyzed.

Six out of the seven sessions for each child were retained for further analysis with the first session being the session containing the first independent steps (FS). The +2 months sessions were excluded due to insufficient data quality or an insufficient amount of data recorded. See Figure 1 for an overview of all included sessions and respective ages and walking ages for P1 and P2.

The mean number of strides included per session was (mean ± std) 133 ± 73 strides for the toddlers’ sessions and a total of 105 strides for the adults (range 14-24 per participant). The FS session for P1 had an exceptionally large number of strides that could be included. To make the number of strides more balanced across sessions, only overground strides with a velocity of more than 0.5 km/h were retained, reducing the total number of strides from 729 to 254 strides for this specific session. Several strides with a Froude number exceeding 1.5 were excluded from the sessions +19 and +32 for both P1 (∼25 strides in total) and P2 (∼60 strides in total) as they were deemed to be sprinting and as such were not comparable to the other data.

**FIGURE 2:**
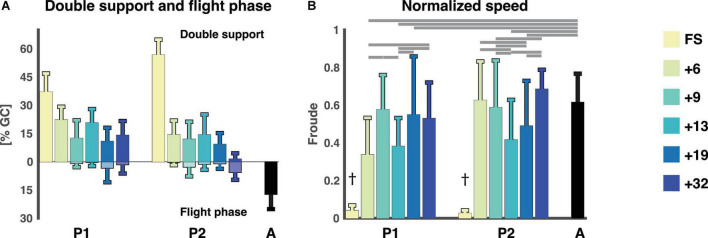
Temporal gait parameters. **(A)** Flight phase and double support phase as a percentage of the gait cycle (mean ± std). Double support phases are on the top with flight phases being the negative percentages below. **(B)** The normalized speed expressed as the Froude number (v^2^/g ⋅ l) for each session and participant (mean ± std). ^†^Denotes a significant difference between current session and all other sessions for that participant as well as adults (*p* < 0.007). Horizontal lines denote significant differences between the Froude numbers for the sessions. A: adults, FS: first steps, %GC: percentage gait cycle, +6, +9, +13, +19, +32 refers to the number of months since onset of independent walking.

The FS sessions had only strides with double support phases whereas the remaining sessions had a mixture of strides with double support and flight phase. The double support and flight phases were expressed as a percentage of the gait cycle. Double support phases were present in all sessions (after the FS sessions) of P1 and P2, with a tendency towards an increased amount of flight phase in the last sessions. The flight phase was shorter than in the adults. The linear regression of the double support phase revealed a significant effect of session, no effect on participant, and only a small interaction effect between session and participant (cf., Figure 2 and Supplementary material 2). The linear regression of the flight phase revealed a significant effect of both session and participant and an interaction effect as well. The normalized speed (Froude number) of the two FS sessions was significantly different from all other sessions for that participant and to that of the adults (*p* < 1 × 10^−14^ for all sessions for both participants). The normalized speeds ranged between 0.34 and 0.69 for the running sessions and three sessions of P1 (+6, +13, and +19, *p* < 2 × 10^−9^) and two sessions of P2 (+13 and +19, *p* < 2 × 10^−7^) were significantly different from the adults, cf. Figure 2.

Like the presence of a flight phase, the Froude number might also not be a good indicator of whether a child is running. The Froude number, the normalized speed, is useful to determine the optimal speed at which to transition from walking to running or vice versa, and in adults this transition occurs at a Froude value of 0.5 (Kram et al., 1997; Gatesy and Biewener, 2009) which the adults exceeded in this study. The mean of the Froude numbers of the toddlers also exceeded 0.5 (P1: 0.58 ± 0.18, 0.55 ± 0.31, 0.53 ± 0.19 for sessions +9, +19, +32 and P2: 0.63 ± 0.21, 0.59 ± 0.25, 0.69 ± 0.10 for sessions +6, +9, +32, respectively) except for +6 (0.34 ± 0.20) and +13 (0.38 ± 0.15) for P1 and +13 (0.42 ± 0.21) and +19 (0.49 ± 0.24) for P2. See above and Figure 2 for statistics. However, it is possible to walk at a Froude value higher than 0.5, it is just not as energetically efficient.

### 3.1. PCA and clustering

**FIGURE 3:**
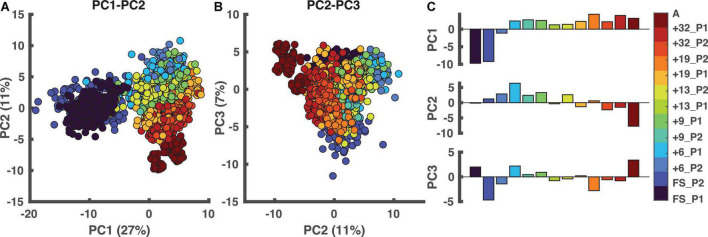
Principal component analysis (PCA). In the two left panels, each dot represents one stride. **(A)** PCA results in PC1-PC2 space. **(B)** The outcome of the PCA in PC2-PC3 space. **(C)** The weightings of all sessions ordered based on walking age, so not ordered per participant. PC1 distinguishes the FS sessions from the other sessions with PC2 distinguishing “mature” from “immature” running. A: adults, PC: principal component, FS: first steps, +6, +9, +13, +19, +32 refers to the number of months since onset of independent walking.

The first three principal components (PCs) accounted for > 45% of the total variance of the data. The scatterplots in Figure 3, detail the spread of data in the 3 PC spaces. We observed that PC1 can distinguish between the FS sessions and the remaining sessions, whereas PC2 seemed to distinguish adult running and later sessions from the early sessions. The loadings associated with these three PCs (Supplementary material 1, 3) were all within the *CI*_95_ except for three. The three parameters not contributing were parameters 74, 79, and 88, i.e., the phase relationship between the two limbs (a measure for interlimb coordination), projection of 1st eigenvector on the shank axis (a measure intersegmental coordination; Borghese et al., 1996; Bianchi et al., 1998; Ivanenko et al., 2007b; Dominici et al., 2010; Bekius et al., 2021), and ratio of left to right leg cycle duration (a measure for intersegmental coordination).

**FIGURE 4:**
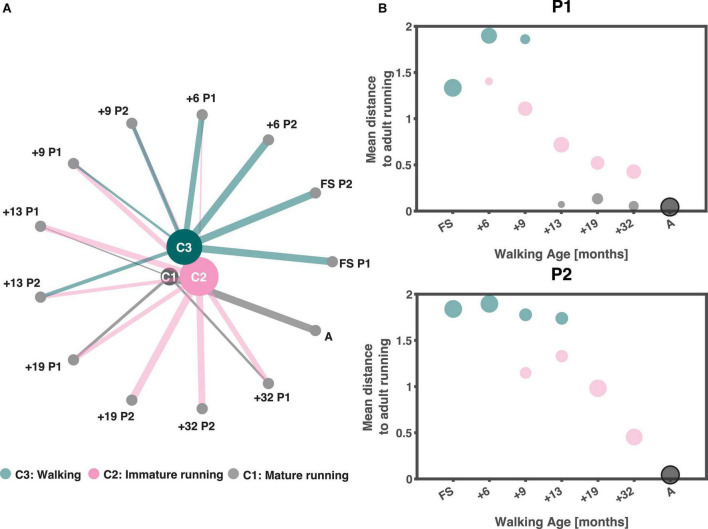
Clustering output. **(A)** Output of clustering ordered based on walking age (time since onset of walking in months) with the youngest session on the right and increasing in walking age in anticlockwise direction. The size of the clusters depends on the number of strides they contain (the larger the node the more strides they contain), similarly are the lines from each node to a cluster a representation of the number of strides (thicker lines equals more strides) from that session that belongs to each cluster larger than 10%. **(B)** Average pairwise correlation distance from each session to those of the adults as a function of walking age (months) for P1 and P2, respectively. Sizing of dots follow the sizing of lines in panel. A: adults, FS: first steps, +6, +9, +13, +19, +32 refers to the number of months since onset of independent walking.

We found three clusters, see Supplementary material 3 for details. The clustering results are depicted in Figure 4A. Every node represents strides from a certain session ordered from lowest to highest walking age from left to right. The lines connecting the sessions to the clusters represent the number of strides larger than ten percent that is present in a certain cluster. The cluster nodes are sized based on the number of strides in each cluster. The three-cluster solution resulted in one cluster that included the adults (“A” on the lower far right of the circle) which could be interpreted as the mature running cluster (C1 cluster), one containing the immature running strides (C2 cluster) and one that included the “walking” strides (C3 cluster). The “walking” cluster contained all strides of the two FS sessions as well as a percentage of strides each from the following sessions (session [% strides]): +6 P1 (81%), +6 P2 (99%), +9 P1 (31%), +9 P2 (52%), +13 P2 (51%). The “immature running” cluster contained some strides from all sessions, except the two FS sessions, +6 P2 and the adults running. Finally, the “mature running” cluster contained all strides from the adults, 32% from +32 P1, 40% from +19 P1, and 16% of the strides from +13 P1. At first glance, P1 and P2 had similar developmental trajectories but a closer look revealed clear differences in that, in contrast to P2, P1 did reach mature running while immature running occurred intermintly within sessions from 9/13 months from onset of independent walking onwards (cf., Figure 4B).

### 3.2. Muscle synergies

The differences in the developmental trajectories shown in Figure 4B may appear quantitatively subtle but – in fact – they are of qualitative nature. P2 shows an improvement in the running maturity which evolves gradually but does not reach full maturity over the observation period. On the other hand, P1 did reach a mature running pattern over the last few recording sessions, but apparently the immature pattern coexisted even once mature running could be accomplished. In searching for the causes underlying these difference, we here dwell more on the result of the analysis of the accompanying muscle synergies. Before going into detail, we would like to note that lateral gastrocnemius (LG) was not analyzed for the FS and +6 sessions of P1 and erector spinae (ES) for +6 of P1. This was due to poor data quality.

**FIGURE 5:**
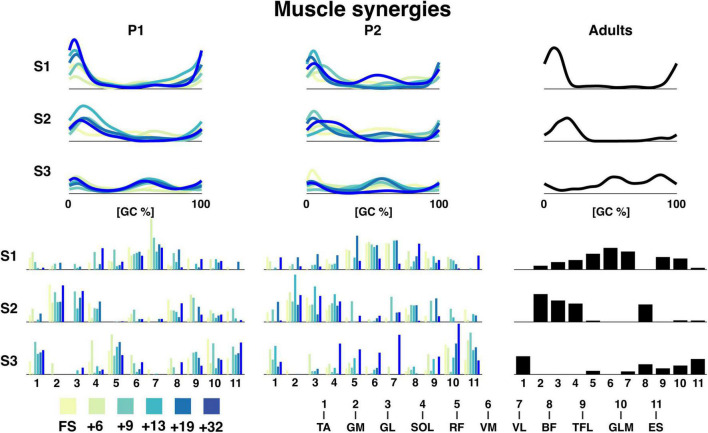
Muscle synergies for P1, P2, and adults. The top graphs are the grand average temporal activation patterns for each session as a function of the gait cycle and the three synergies. Amplitude is in arbitrary units. The lower bar graphs are the weighting coefficients for the muscle synergies. The naming of the muscles can be seen below. TA: tibialis anterior, GM: gastrocnemius medialis, GL: gastrocnemius lateralis, SOL: soleus, RF: rectus femoris, VM: vastus medialis, VL: vastus lateralis, BF: biceps femoris, TFL: tensor fascia latae, GLM: gluteus maximus, ES: erector spinae, +6, +9, +13, +19, +32 refers to the number of months since onset of independent walking.

The reconstruction accuracy (RA) revealed different numbers of muscle synergies between sessions with median of three muscle synergies (IQR: 3:4.5) across all sessions. The RA was 64.2 ± 1.8, 64.1 ± 2.6, and 66.3 for P1, P2, and the adults, respectively, for three synergies. The temporal activation patterns of the first of the three synergies had the most activity at foot contact, the weight acceptance phase, with the knee extender muscles (RF, VM, VL, and RF muscles) being the predominant influencers both for toddlers and adults with some contribution also of the TFL and GLM muscles (cf. Figure 5). The second components were related to end of stance, the propulsion phase, with the largest contributions of MG, LG, and SOL. The third synergy was more variable with activity from TA and ES with most activity at swing. Synergies were comparable across sessions for P1 and P2. For P1, the most notable development in the muscle synergies in time after onset of independent walking, was the increase in amplitude, especially in synergy 1. This increase in amplitude across age/time since onset of independent was not clear to the same extent in P2.

**FIGURE 6:**
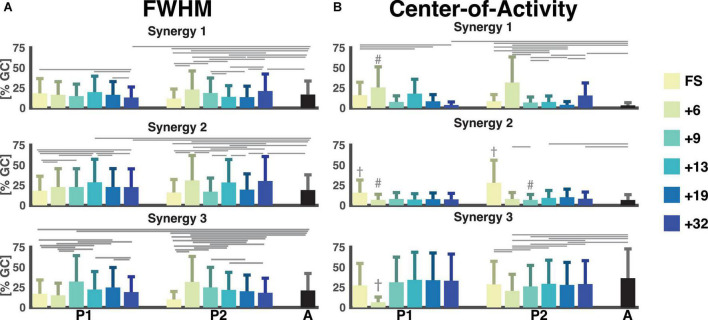
Full-width half-maximum and center-of-activity of muscle synergies. Color notation as to the right. **(A)** The full-width half-maximum (FWHM) was calculated for the main peak of each stride for each muscle synergy. It is represented as a percentage of the gait cycle. **(B)** The center-of-activity (CoA) were was expressed as the percentage of the gait cycle. The horizontal lines represent a significant difference, *p* < 0.007 within a participant and the adults. The dagger (†) represents a significant difference between session and all other sessions. A hash (#) represents a significant difference between session and all later sessions and adults. GC: gait cycle, FWHM: full-width at half-maximum, A: adults, FS: first steps, +6, +9, +13, +19, +32 refers to the number of months since onset of independent walking.

FWHM and CoA mark the ability to quickly contract a muscle and the timing of the muscle contraction, respectively. The FWHM of the temporal patterns of the three synergies was comparable across sessions with a large variance between strides (cf. Figure 6). The most pronounced differences were found between the FWHM of the sessions of P2 and the adults (e.g., FS, +6, +13, +19, and +32 all had a *p* < 0.0001 for synergy 1, +6, +13, +32 were all *p* < 0.001 for synergy 2) with some in-between significant differences between the sessions within P1 and P2. Synergy 3 of P2 had a characteristic pattern of a reduction of the FWHM from the first running session (+6) to the last running session (+32) with significant *p*-values of *p* < 0.0001 for the +6 session compared to +19, +32, and adults and *p* = 0.0067 for the +6 session compared to the +13 session.

The CoA of the synergies were way more variable in the children than in the adults. In the latter the CoAs were within the same 25% of the gait cycle across strides (between 0 and 25% of the gait cycle for synergy 1 and 2, and within 63 and 83% of the gait cycle for synergy 3). For the first and third pattern, the range of the CoA for P1 and P2 covered all percentages of the gait cycle for all sessions. Most noteworthy is the mean CoA for the second temporal pattern which occurred later in the FS sessions (31.6% ± 18.5% and 56.3% ± 18.7% of the gait cycle) for P1 and P2, respectively, compared to the mean over the other sessions, which ranged from 13.8% to 16.0% for P1 and 13.6% to 20.2% for P2 (*p* < 6 × 10^−4^ and *p* < 2 × 10^−16^, respectively). Here, the adult patterns were similar with a mean of 13.3% ± 3.6% of the gait cycle for the second component.

## 4. Discussion

### 4.1. PCA and clustering – Classifying running maturity

We succeeded to classify the development of running maturity over the span of six recording sessions of almost three years in two toddlers matched on walking age. That is, our shotgun approach combining PCA and hierarchical clustering enabled us to estimate the maturity of very early development of running.^1^ After walking the first independent steps, our participants developed the capacity of running, though to different degrees of maturity. It appears that mature running patterns can coexist next to immature ones, which occur earlier in the course of developement.

As said, in particular in P1, several sessions had strides in two different clusters. Since the data were collected not only during overground running but also on the treadmill it might have been that the more mature strides were recorded overground and the more immature ones on the treadmill. A lack of treadmill experience may hence have cause the ‘return’ to the immature mode of locomotion. However, analysis of the strides falling into the more mature or immature clusters did not reveal such a pattern, see Supplementary material 3.

Remarkably, the FS session of P1 was relatively close to mature running in terms of the average pairwise distance to the adults. While this might hint at shortcomings of our clustering approach (see also below), already in PC1-PC2 space the first step session (very dark blue) was indeed close to the adults. In a future study we will compare new walkers to very experienced runners. There we will also include adult walking, i.e., experienced walking, and expect that the FS strides will becloser to this cluster than to adult running.

In the current two children we cannot pinpoint a moment when the running pattern differs from the walking pattern and becomes like the adult running pattern. This is mostly due to a substantial variability between and within sessions in the majority of outcome variables. We would like to stress anyway that we were able to show that the two toddlers seemingly display different paths towards running maturity.

### 4.2. Muscle synergies – What underlies running maturity?

We determined three muscle synergies. This number is smaller than in many other studies on running where the number is usually four or more (Cappellini et al., 2006; Santuz et al., 2018b; Cheung et al., 2020; Bach et al., 2021b). We chose to determine the number of muscle synergies based on finding the plateau of the reconstruction accuracy (RA) using the linear-fit method introduced by Cheung et al. (2005). Whether or not this explains this discrepancy with the literature is unclear. A recently method proposed by Ballarini et al. (2021) where the consistency and similarity of both activation patterns and weighting coefficients were determined may shed light on this. However, most methods of determining the number of synergies require setting some threshold which leaves this an open issue for future research (Sylos-Labini et al., 2022); see Supplementary material 5 for an alternative to the reconstruction accuracy (RA) used here.

Synergy 2 shows a phase shift as evidenced in the CoA from 30-50% of the gait cycle for the FS session to around ∼15% of the gait cycle for the later sessions. The phase shift occurs from the +6 session and is stable across the running sessions, which indicates that a contraction of the foot flexors matching the shorter stance phase is important for even early running patterns.

The analysis of FWHM of the temporal activation patterns of the muscle synergies allowed us to quantify the duration of activity of the muscles contributing to this particular synergy. FWHM of motor primitives or muscle synergies has previously been hypothesised to be a measure for robustness of the motor control (Martino et al., 2014; Martino et al., 2015; Santuz et al., 2018a; Mileti et al., 2020; Santuz et al., 2020). For both synergy 1 and 2, we found the most differences from the adult patterns to the patterns of P2 and not P1, which indicates similar widening between P1 and the adult pattern already from an early running pattern. It is likely that this trend was indeed an underlying reason for why the running patterns of this participant has been considered to mature earlier than P2. It is likely that this trend was indeed an underlying reason for why the running patterns of this participant has been considered to mature earlier than P2. We also observed that for both synergy 1 and 2 that there was first an increase followed by a reduction in the FWHM from the +13 session. This is the same timepoint at which the very mature strides appeared. In P2, the +6 and +32 sessions for synergy 1 both differed from most of the other sessions with a decrease in the FWHM from the +6 session to the +19 session with the +6 and +32 sessions being similar. The decrease from 6 months since onset of independent walking until +19 months could be due to an improved ability to narrow the duration of the activation pattern and reduce the overlap of the muscle synergies at the weight acceptance phase, where the increase in the FWHM at 32 months could be explained by a possible altered running pattern that is not yet finetuned.

### 4.3. Methodological choices and limitations

The EMG was processed by normalizing the amplitude to the mean of the data across strides. According to Besomi et al. (2020), a normalization to the mean can lead to a reduction in the inter-individual variation in amplitude. Obtaining a more optimal normalization via maximal voluntary contraction may, however, not be feasible, especially in young children. Yet, we must admit that normalization to the mean might have resulted in some unwarranted high weighting for some muscles (Besomi et al., 2020).

The main limitation of this study is the lack of power by only having two participants. Without a doubt this limits the ability to generalize our results. Despite the lack of generalizability, we consider it a valuable starting point, first in methods and – more importantly, in clarifying that time since onset of independent walking does not appear to be a solid indicator of the maturity of running patterns in very young children.

The two clusters, C3 “walking” and C2 “immature running” were so close to each other that they are just overlapping in terms of our measure of maturity, the mean pairwise correlation distance to that of the adults. This may suggest that a cluster solution with three clusters may not have been optimal. However, the mean pairwise distance to the adults did not change with an altered number of clusters. We are hence convinced that our measure can be used for determining the “order of maturity”. On the other hand, the FS session of P1 can be considered more mature than +6 and +9, two sessions containing running strides. Apparently, the distance measure and linkage method used to create the dendrogram was less optimal than in our previous study (Bach et al., 2021a). However, a combination of other distance measures and linkage methods did not yield better cophenetic correlation coefficients (CCC). The only a Euclidian distance measure with either average (CCC: 0.83) or centroid algorithms (CCC: 0.84) showed comparable results but cause problems, e.g., dendrogram with non-monotonic links.

Future research should be focused on investigating larger number of children. When doing so, we advocate combining many kinematic and neuromuscular parameters to fully investigate the development of the running patterns in very young children.

## 5. Conclusion

Our study is unique in that the development of running was monitored longitudinally over a three-years span with highly frequent assessments of kinetics, kinematics, and electromyography. It provides a first view on the effect of time since onset of independent walking on the development of running. Running development followed different trajectories that we quantified ‘blindly’ via a shotgun approach after combining various biomechanical and neuromuscular parameters. Evidently, the development of running can take different trajectories including the co-existence of immature and mature running within the same session in a child. Muscle synergy analysis may help explaining why the development can differ between children, though there is a long way to clarifying this with statistical robustness.

## Data availability statement

The raw data supporting the conclusions of this article will be made available by the authors, without undue reservation.

## Ethics statement

The studies involving human participants were reviewed and approved by the Scientific and Ethical Review Board of the Faculty of Behavioural and Movement Sciences, Vrije Universiteit Amsterdam, Netherlands (File number: VCWE-2016-082) and the Ethics Committee of the Santa Lucia Institute, Rome, Italy (Prot. CE-AG4-PROG.99-155). Written informed consent to participate in this study was provided by the participants’ legal guardian/next of kin. Written informed consent was obtained from the minor(s)’ legal guardian/next of kin for the publication of any potentially identifiable images or data included in this article.

## Author contributions

MB: conceptualization, formal analysis, investigation, data curation, writing – original draft, writing – review and editing, and visualization. CZ: conceptualization, investigation, and writing – review and editing. GC and FL: investigation and writing – review and editing. YI: investigation, funding acquisition, and writing – review and editing. AD: writing – review and editing, supervision, and visualization. ND: conceptualization, formal analysis, investigation, writing – review and editing, supervision, and funding acquisition. All authors contributed to the article and approved the submitted version.

## Footnotes

1 While this – in principle – supports the combination of kinetic, kinematic, and electromyographic data in a single ‘shotgun’ approach, it leaves the question of which factors or parameters are causing the (differences in) development open. However, the parameters dominating the PCA can be readily determined. We briefly illustrate this by investigating whether a reduced number of parameters may lead to results similar to the ones reported in the body text. For this sake we retained only loadings contributing > 0.6 and repeated the cluster analysis. In total 46 parameters were included in the second analysis (see Supplementary material 4). These cluster results differed from our main ones in that more sessions/strides were considered “mature” running and more strides/sessions were considered “immature” running instead of walking. Thus, clustering seems less sensitive to individual differences in the strides with a reduced number of parameters. Put differently, parameters not contributing more than 60% to the PCA are still relevant when clustering.
