# Supplementary material for: Development of running is not related to time since onset of independent walking, a longitudinal case study
Source: Front Hum Neurosci. 2023 Feb 16;17:1101432. doi: 10.3389/fnhum.2023.1101432 (PMC9978154; doi:10.3389/fnhum.2023.1101432)
Supplement: Supplementary file 1 [file Data_Sheet_1.PDF]

Supplementary Material

1 Supplementary Material 1

List of parameters and their normalizations before being z-scored and included into the PCA.

Table S1: Included parameters in the principal component analysis.

PARAM	DETAILED EXPLANATION	UNIT [NORM]
**TEMPORAL FEATURES**		
1	Stride duration	s
2	Froude velocity	
3	Stance duration	s
4	Percentage swing duration	% GC
5	Percentage stance duration	% GC
6	Percentage double support	% GC
7	Percentage flight phase	% GC
8	Stride length (1D)	[1/l]
9	Stride length (3D)	[1/l]
**LIMB ENDPOINT (VM) TRAJECTORY**		
10	Step length	[1/l]
11	Step height	[1/l]
12	Maximum backward position	[1/l]
13	Maximum forward position	[1/l]
14	Maximum velocity during swing	m/s
15	Relative timing of max velocity during swing	% GC
16	Acceleration at swing onset	m/s^2
17	Endpoint velocity	m/s
18	Orientation of velocity vector at swing onset	rad
19	Position of ankle with respect to hip at FC	[1/l]
20	Position of ankle with respect to hip at FO	[1/l]
21	Position of ankle with respect to hip at SE	[1/l]
**STABILITY**		
22	Lateral displacement of foot during swing	[1/l]
23	Step length	[1/l]
24	Step width (ML)	[1/l]
25	Hip midpoint variability (ML)	
26	Hip midpoint variability (vert)	
27	Variability of sagittal trunk oscillations	
28	Variability in vel. of sagittal trunk oscillations	
29	Variability of Medio-lateral hip rotations	
30	Amplitude of trunk Medio-lateral movement	deg
31	Amplitude of trunk Vertical movement	deg
32	Variability of Medio-lateral trunk movement	
33	Variability of Vertical trunk movement	
**JOINT AND SEGMENTAL ANGLES**		
34	Hip elevation angle (min)	deg
35	Thigh elevation angle (Min)	deg
36	Shank elevation angle (Min)	deg
37	Foot elevation angle (Min)	deg
38	Main-leg elevation angle (Min)	deg
39	Hip elevation angle (Max)	deg
40	Thigh elevation angle (Max)	deg
41	Shank elevation angle (Max)	deg
42	Foot elevation angle (Max)	deg
43	Main-leg elevation angle (Max)	deg
44	Hip joint angle (Min)	deg
45	Knee joint angle (Min)	deg
56	Main-leg elevation angle (Amp)	deg
57	Hip joint angle (Amp)	deg
58	Knee joint angle (Amp)	deg
59	Ankle joint angle (Amp)	deg
60	Main-leg medio-lateral angle (Amp)	deg
**LEG/JOINT ANGULAR VELOCITY**		
61	Main-leg velocity (Min)	deg/s
62	Hip joint velocity (Min)	deg/s
63	Knee joint velocity (Min)	deg/s
64	Ankle joint velocity (Min)	deg/s
65	Main-leg velocity (Max)	deg/s
66	Hip joint velocity (Max)	deg/s
67	Knee joint velocity (Max)	deg/s
68	Ankle joint velocity (Max)	deg/s
69	Main-leg velocity (Amp)	deg/s
70	Hip joint velocity (Amp)	deg/s
71	Knee joint velocity (Amp)	deg/s
72	Ankle joint velocity (Amp)	deg/s
**INTRA-LIMB COORDINATION**		
73	Correlation between the two limbs AP direction	
74	Phase relationship between the two limbs	
**INTERSEGMENTAL COORDINATION**		
75	Percentage of variance (1st u)	
76	Percentage variance (2nd u)	
77	Percentage variance (3rd u)	
78	Projection of 1st u on thigh axis	
79	Projection of 1st u on shank axis	
80	Projection of 1st u on foot axis	
81	Projection of 2nd u on thigh axis	
82	Projection of 2nd u on shank axis	
83	Projection of 2nd u on foot axis	
84	Projection of 3rd u on thigh axis	
85	Projection of 3rd u on shank axis	
86	Projection of 3rd u on foot axis	
87	Area of the gait loop	deg^2
88	Ratio of left to right leg cycle duration	
**INTERLIMB COORDINATION**		
89	Phase difference hip and thigh elev. angles	
90	Phase difference thigh and shank elev. angles	
91	Phase difference shank and foot elev. angles	
92	Max r (hip and thigh elevation angles)	
93	Max r (thigh and shank elevation angles)	
94	Max r (shank and foot elevation angles)	
95	Max r (hip and knee joint angles)	
96	Max r (knee and ankle joint angles)	
**PENDULUM/SPRING MECHANISM**		
97	Amplitude of vertical hip displacement	m
98	Amplitude of ML hip displacement	m
**TRUNK SEGMENTAL AND JOINT ANGLES**		

46	Ankle joint angle (Min)	deg	99	Trunk elevation angle (Min)	deg
47	Main-leg abduction (Min)	deg	100	Trunk elevation angle (Max)	deg
48	Hip joint angle (Max)	deg	101	Trunk elevation angle (amp)	deg
49	Knee joint angle (Max)	deg	**MUSCLE SYNERGIES**		
50	Ankle joint angle (Max)	deg	102	Full-width half-maximum – Synergy 1	% GC
51	Main-leg abduction (Max)	deg	103	Full-width half-maximum – Synergy 2	% GC
52	Hip elevation angle (Amp)	deg	104	Full-width half-maximum – Synergy 3	% GC
53	Thigh elevation angle (Amp)	deg	105	Center of activity – Synergy 1	% GC
54	Shank elevation angle (Amp)	deg	106	Center of activity – Synergy 2	% GC
55	Foot elevation angle (Amp)	deg	107	Center of activity – Synergy 3	% GC

Param: parameter; Norm: normalization; l, leg-length; FC: foot contact, FO: foot off, SE: swing end; AP: anterior-posterior; ML: medio-lateral, W: body weight; SS: single support; d: stride length; min: minimum; max: maximum; amp: amplitude; u: eigenvector.

2 Supplementary Material 2

To quantify the influence of the session and participants on the ability to run with either a double support (DS) phase or a flight phase (FP), a linear regression model was fitted to all data with either DS or FP as the response variable and the session, participant and the interaction between session and participant as predictors. $p < 0.05$ were considered significant for this purpose. We fitted a least squares exponential function of the dependence of Froude on the ability to run with a flight phase and double support phase, respectively and report the adjusted R^2 values. A two-sample t-test was performed on the Froude values for the treadmill strides and overground strides for each session.

Table S2: Statistics on double support phase and flight phase

	Factor	*Estimate*	*SE*	*t*	*p-value*
DS	Intercept	42.62	1.70	25.11	$< 2 \times 10^{-16}$
	Session	-4.84	0.35	-13.98	$< 2 \times 10^{-16}$
	Participant	-1.83	1.09	-1.68	0.09
	Session:Participant	-0.47	0.20	-2.31	0.02
FP	Intercept	3.42	0.65	5.29	$< 2 \times 10^{-7}$
	Session	-1.27	0.13	-9.61	$< 2 \times 10^{-16}$
	Participant	-3.31	0.42	-7.98	$< 2 \times 10^{-16}$
	Session:Participant	1.45	0.08	18.64	$< 2 \times 10^{-16}$

DS: Double support phase, FP: Flight phase, SE: Standard error, t: t-statistics

The Froude value for strides on the treadmill were in all cases significantly lower than the Froude value for strides recorded while locomoting overground as can be seen in Table S3. Not all sessions contained strides recorded during both modalities.

Table S3: Comparisons of the Froude values for treadmill and overground strides

Participant	*Froude – OVG*	*Froude - TM*	*p-value*
FS P1	0.04		$< 2 \times 10^{-16}$
FS P2	0.02	0.04	9.5×10^{-6}
+6 P1	0.34		
+6 P2	0.63		
+9 P1	0.58		
+9 P2	0.65	0.45	1.7×10^{-5}
+13 P1	0.59	0.32	$< 2 \times 10^{-16}$
+13 P2	0.51	0.25	3.3×10^{-16}
+19 P1	0.86	0.41	1.5×10^{-15}
+19 P2	1.04	0.40	$< 2 \times 10^{-16}$
+32 P1	1.06	0.50	$< 2 \times 10^{-16}$
+32 P2	1.03	0.68	$< 2 \times 10^{-16}$
A		0.62	

Missing values are due to no strides recorded for that modality for that session.
A: Adults, OVG: Overground, TM: Treadmill, FS: First steps

There was a strong inverse relationship between the Froude value and the DS phase ($R^2 = 0.79$) meaning that the lower the Froude value, the more DS was present (cf., Figure S1). However, there no significant relationship ($R^2 = 0.27$) between the Froude value and the amount of FP and there is almost no flight phase present with a Froude value between 0 and 0.4. The strong presence of a relationship between the double support phase and the normalized speed, but the lack of the same relationship between the flight phase and the normalized speed indicates that for running the ability to run with a flight phase is not merely a function of running at a certain speed, but more factors influence this ability.

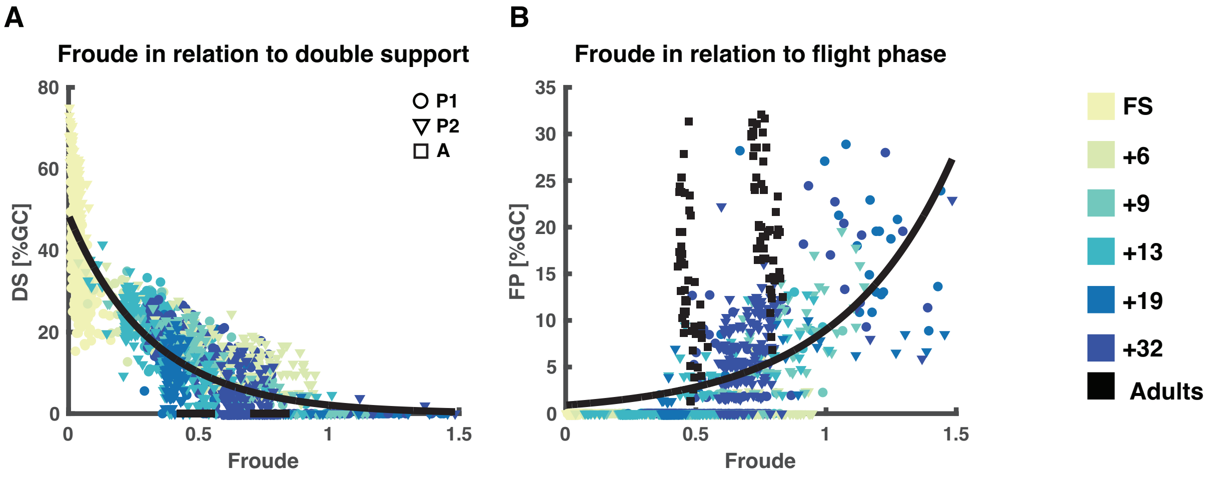

Figure S1: Relationship between Froude and DS and FP. A) Relationship between double support expressed as a percentage of the gait cycle as a function of the Froude value. A least-squares exponential function was fitted and resulted in an adjusted $\boldsymbol{R^2}$ = 0.79 which means a decrease in double support is correlated to an increase in dimensionless speed. B) Relationship between flight phase expressed as a percentage of the gait cycle as a function of the Froude value. A least-squares exponential function was fitted and resulted in an adjusted $\boldsymbol{R^2}$ = 0.27 which means no relation between the amount of flight phase and dimensionless speed. DS: Double support, FP: Flight phase, %GC: percentage gait cycle, +6, +9, +13, +19, +32 refers to the number of months since first steps, i.e., the walking age.

3 Supplementary Material 3

Loadings explaining the contribution to each PCA. First parameter to the right, parameter 107 on the left. See Supplemental Material 1 for overview of the different parameters. The darker the color (red or blue) the higher the contribution to the relevant PC.

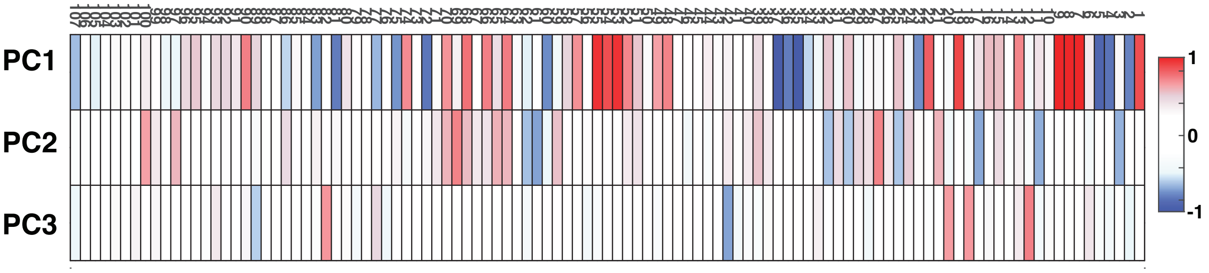

Figure S2: Loadings for PC1-PC3. Color coding refers to the contribution of each loading. The darker the color the higher the contribution. See Table S1 for overview of all 107 parameters.

We first built a dendrogram using average links (unweighted pair group method with arithmetic mean) of the first three PCs. The cophenetic correlation coefficient (CCC) for the correlation distance measure with average links was 0.84 which was comparable or higher than other combinations of distance measure and linkage methods. Combining a visual inspection of the dendrogram with the Calinzki-Harabasz stopping rule, resulted in three clusters. The Calinzki-Harabasz stopping rule run from 1-10 clusters revealed a cluster solution of two to be the most optimal, however the visual inspection resulted in three clusters to also allow for a possible separation, not only between walking in the FS sessions and the running in the other sessions but also between immature and mature running.

Dendrogram describing the split of clusters. The y-axis is a measure for the distance between clusters as measured by the correlation distance. The taller the links between two lead nodes, the longer the leaf nodes (or clusters) are located from each other in 3D space.

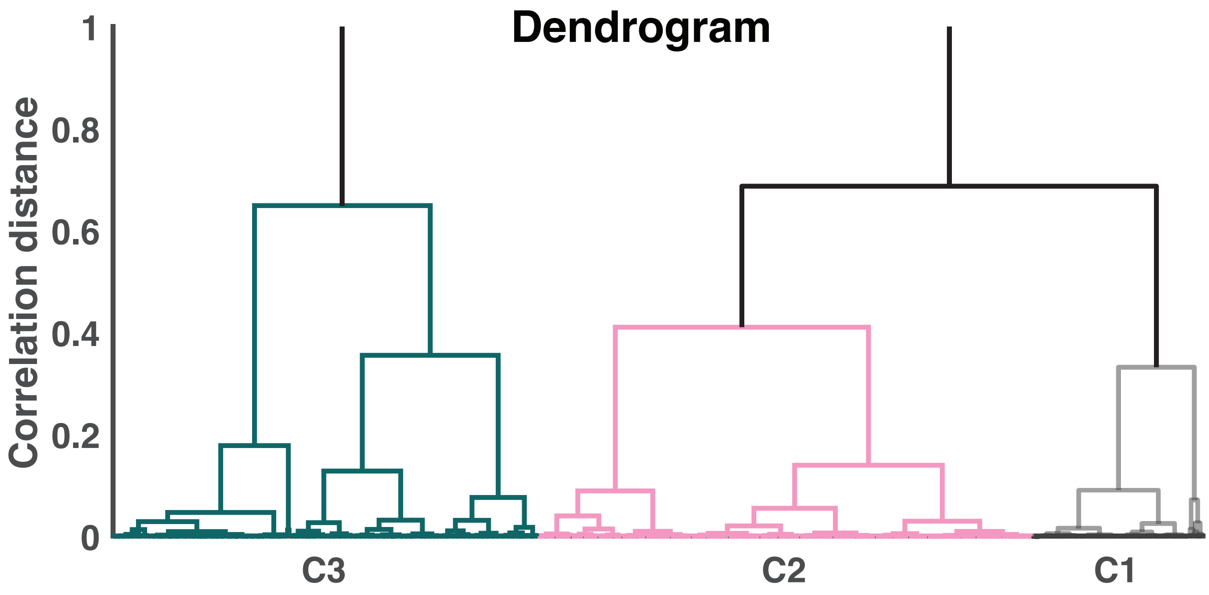

Figure S3: Dendrogram for clustering results.

Table S4 represents the strides that belong to each cluster for treadmill and overground locomotion, respectively. What is clear in this overview is that the modality (treadmill or overground locomotion) does not account for the maturity of the strides.

Table S4: Overview of overground and treadmill strides for each cluster

Participant	Cluster 1		Cluster 2		Cluster 3		Total strides
	OVG	TM	OVG	TM	OVG	TM	
FS P1					254		254
FS P2					77	22	99
+6 P1			9		38		47
+6 P2			1		108		109
+9 P1	1		19		9		29
+9 P2	2	5	31	29	64	8	139
+13 P1	1	15	16	63	6	1	102
+13 P2			35	43	68	14	160
+19 P1	13	29	18	45	2		107
+19 P2		2	12	78	1		93
+32 P1	9	69	6	163			247
+32 P2		23	5	209	2		239
A		103		2			105

Missing values are due to no strides recorded for that modality for that session. Red values represent strides that are not included in the cluster results as they account for less than 10% of the total number of strides. A: Adults, OVG: Overground, TM: Treadmill, FS: First steps

4 Supplementary Material 4

To investigate if a smaller number of parameters lead to similar results as the full analysis a second analysis was done with the loadings contributing >0.6 to either of the three PCs. This reduced number of loadings means that 46 parameters were analyzed in total. See Table S5 below for parameters.

Table S5: List of parameters included into PCA in reduced analysis.

PARAM	DETAILED EXPLANATION	NEW PARAM
**TEMPORAL FEATURES**		
1	Stride duration	1
2	Froude velocity	2
3	Stance duration	3
4	Percentage swing duration	4
5	Percentage stance duration	5
6	Percentage double support	6
8	Stride length (1D)	7
9	Stride length (3D)	8
**LIMB ENDPOINT (VM) TRAJECTORY**		
10	Step length	9
12	Maximum backward position	10
13	Maximum forward position	11
14	Maximum velocity during swing	12
18	Orientation of velocity vector at swing onset	13
19	Position of ankle with respect to hip at FC	14
20	Position of ankle with respect to hip at FO	15
21	Position of ankle with respect to hip at SE	16
**STABILITY**		
23	Step length	17
24	Step width (ML)	18
28	Variability in vel. of sagittal trunk oscillations	19
**JOINT AND SEGMENTAL ANGLES**		
36	Shank elevation angle (Min)	20
37	Foot elevation angle (Min)	21
38	Main-leg elevation angle (Min)	22
43	Main-leg elevation angle (Max)	23
49	Knee joint angle (Max)	24
50	Ankle joint angle (Max)	25
53	Thigh elevation angle (Amp)	26
**54**	Shank elevation angle (Amp)	27
**55**	Foot elevation angle (Amp)	28
**56**	Main-leg elevation angle (Amp)	29
**58**	Knee joint angle (Amp)	30
**LEG/JOINT ANGULAR VELOCITY**		
61	Main-leg velocity (Min)	31
62	Hip joint velocity (Min)	32
65	Main-leg velocity (Max)	33
67	Knee joint velocity (Max)	34
69	Main-leg velocity (Amp)	35
70	Hip joint velocity (Amp)	36
71	Knee joint velocity (Amp)	37
**INTRA-LIMB COORDINATION**		
73	Correlation limb-arm AP direction	38
**INTERSEGMENTAL COORDINATION**		
75	Percentage of variance (1st u)	39
76	Percentage variance (2nd u)	40
78	Projection of 1st u on thigh axis	41
82	Projection of 2nd u on shank axis	42
83	Projection of 2nd u on foot axis	43
84	Projection of 3rd u on thigh axis	44
**INTERLIMB COORDINATION**		
91	Phase difference shank and foot elev. angles	45
**TRUNK SEGMENTAL AND JOINT ANGLES**		
101	Trunk elevation angle (amp)	46

Param: parameter; Norm: normalization; l, leg-length; FC: foot contact, FO: foot off, SE: swing end; AP: anterior-posterior; ML: medio-lateral, W: body weight; SS: single support; d: stride length; min: minimum; max: maximum; amp: amplitude; u: eigenvector.

The three PCs explained more than 70% of the variance of the original dataset (PC1: 48%, PC2: 14%, and PC3: 10%). All parameters included contributed more than the 95%CI. PC1 distinguished the first sessions from the other sessions with no clear effect of PC2 and PC3.

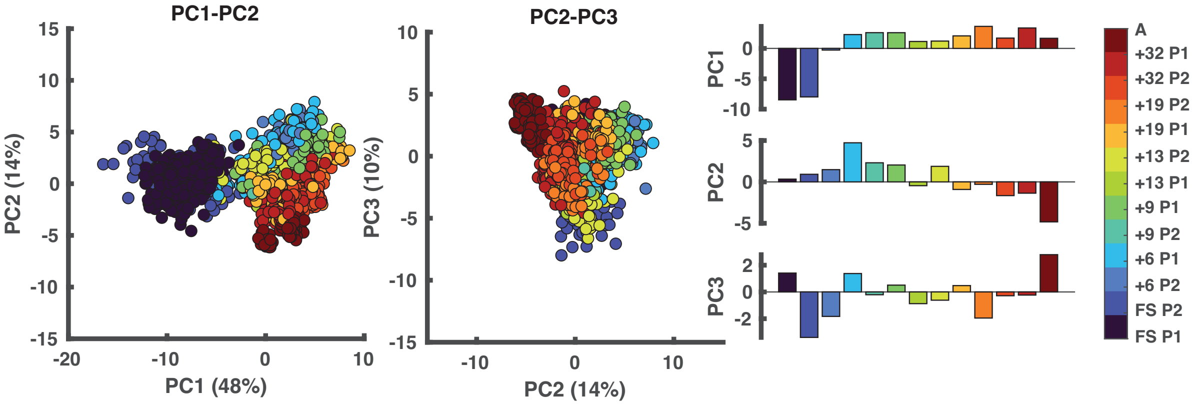

Figure S4: Output of Principal Component Analysis (PCA) on 46 parameters. A) PC1-PC2 space. B) PC2-PC3 space. Color coding as to the right. Each dot represents one stride.

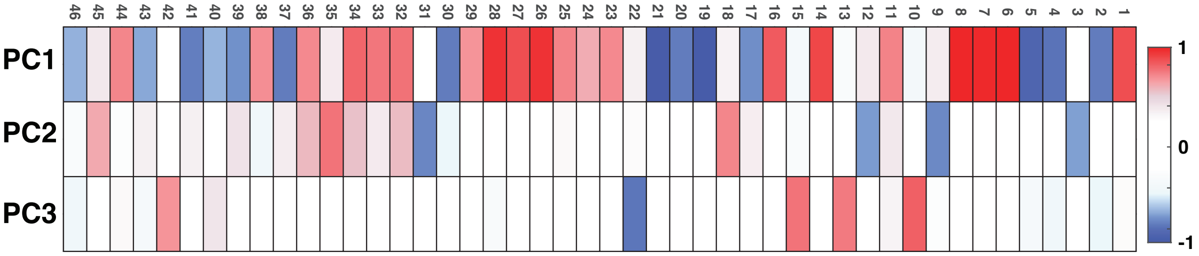

Figure S5: Loadings for PC1-PC3. Color coding refers to the level of contribution with the darkest colors providing the highest loadings. For information on which parameters are which, refer to Table S5.

The loadings for the three PCs were all high for PC1 and they all contributed more than 0.6 to any of the three PCs.

The clustering was done in similar way as the original analysis with a correlation distance and average linkage. A dendrogram was created and the cophenetic correlation coefficient was 0.80 for this cluster solution, see Figure S6.

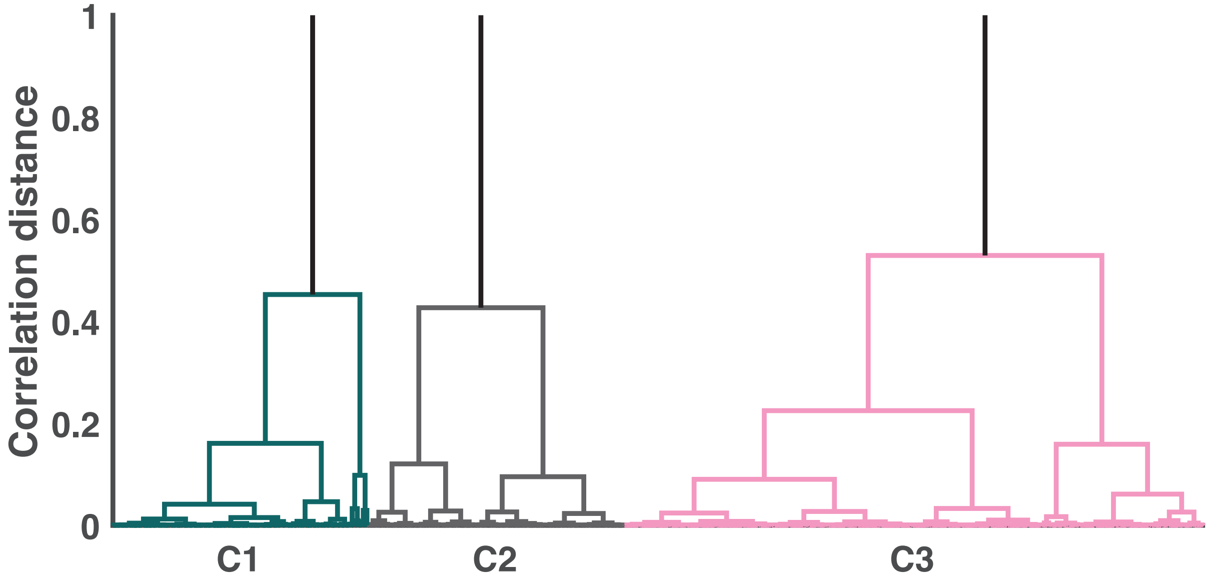

Figure S6: Dendrogram for reduced analysis. Colours represent each their cluster. The y-axis of the plot refers to the correlation distance. Cluster 1 (C1) and cluster2 (C2) are closest to each other with C3 being on the far right.

The Calinzki-Harabasz rule resulted in an optimal number of 2 clusters, however, the visual inspection of the dendrogram revealed that three clusters resulted in the least inconsistent links and so the three-cluster solution was used – this also made the supplemental analysis comparable to the main analysis.

The cluster solution with only a subset of the parameters resulted in a slightly different solution of the clusters than the main analysis. More sessions are now clustered in the adult cluster, i.e., the "mature running" cluster (c.f., Figure S7A). This applies to +19 of P2 and more strides from +13 of P1 also now fall in the mature cluster. Similarly, are there fewer strides/sessions falling into the "walking" cluster such as the two +6 and +9 sessions. This indicates that a reduced number of parameters means that the clustering is less sensitive to the individual differences in the strides. The trend that P1 has a faster development toward a mature pattern than P2 is still present in this analysis.

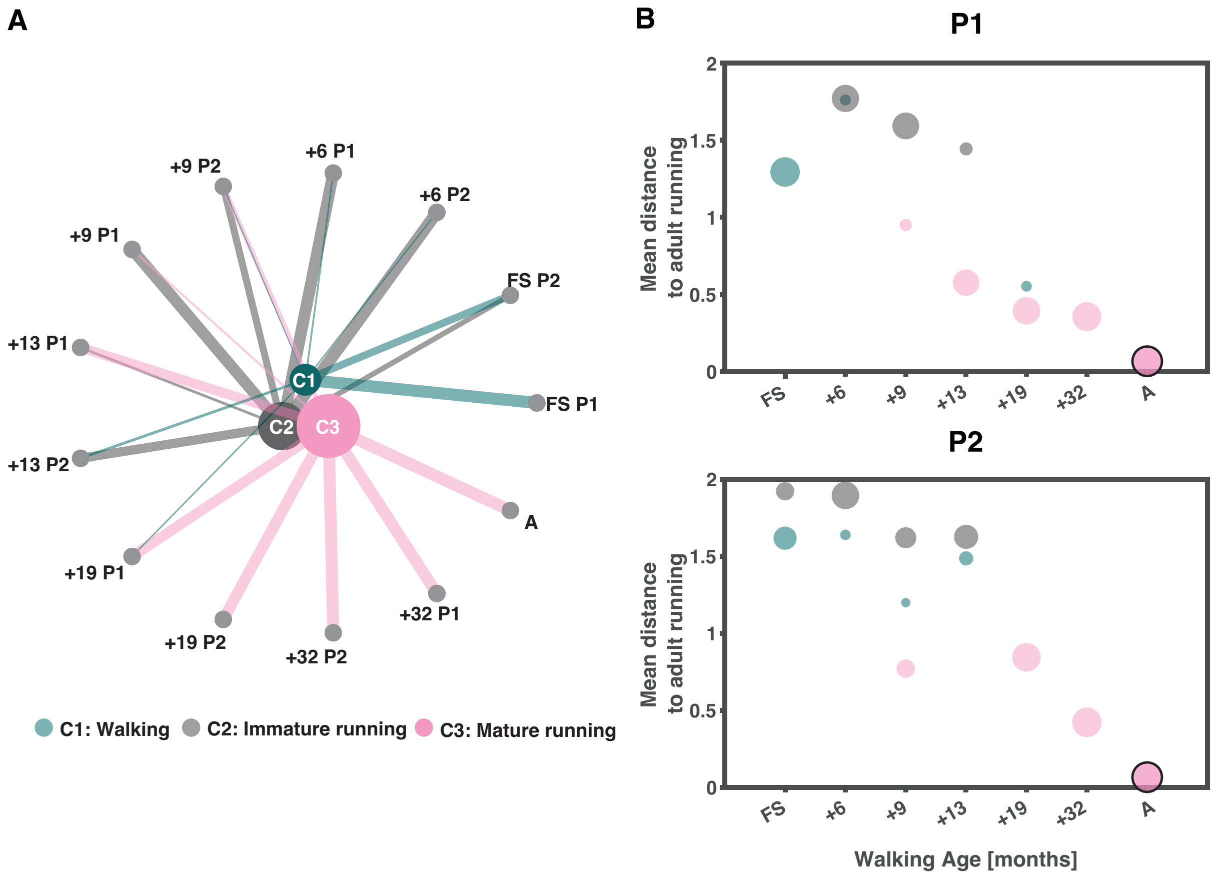

Figure S7: Output of clustering. A) Output of clustering ordered based on walking age (months) with the youngest session on the right and increasing in walking age in anticlockwise direction. The size of the clusters depends on the number of strides they each contain (the larger the cluster node the more strides they contain), similarly are the lines from each node to a cluster a representation of the number of strides from that session that belongs to each cluster larger than 10%. B) Calculated average pairwise correlation distance from each session to those of the adults as a function of walking age (months). Sizing of dots follow the sizing of lines in panel A. C) calculated average pairwise correlation distance to the adults as a function of maturity. Note that some sessions contribute to more than one cluster and thus are repeated on the upper x-axis. The color notation is similar as in (A) and (B). D) Output of clustering based on maturity of the gait patterns with the most immature gait pattern on the far right with increasing maturity in anticlockwise direction. FS: First steps

That all parameters contributed more than 0.6 to the PCA indicates that they are all relevant for the analysis and thus are a good starting point if one wants to pinpoint the exact parameters influencing the maturity of gait patterns in very young children.

5 Supplementary Material 5

The reconstruction accuracy (RA) has the advantage of exploiting the Frobenius norm which is the optimization method also used for the (W)NMF. However, the VAF method is more widely used, so we also compared the RA to the VAF for the chosen number of synergies. We determined VAF as the mean-uncentered VAF.

$$VAF = 1 - \frac{\|EMG - W \cdot H\|^2}{\|EMG\|^2}$$

where W and H represent the weighting coefficients and activation patterns of the synergies, respectively. When using VAF, a common method to determine the number of synergies needed is that they should explain a minimum of 80-90% of the data. Three synergies explain a mean of 87.16% ± 1.29%, 87.07% ± 1.89%, and 88.66% for P1, P2, and adults, and the next added synergies only add 3.34% ± 0.40%, 3.79% ± 1.04%, and 3.14%, respectively. Thus, despite the number of synergies being lower than in other studies, the three synergies still explain a large portion of the original data and adding another synergy does not add any substantial variance to the data.
